# Experiences of Leaders in Diversity, Equity, and Inclusion in US Academic Health Centers

**DOI:** 10.1001/jamanetworkopen.2024.15401

**Published:** 2024-06-13

**Authors:** Caitlin J. Esparza, Mark Simon, Maya R. London, Eraka Bath, Michelle Ko

**Affiliations:** 1 Department of Obstetrics, Gynecology and Reproductive Sciences, University of California, San Francisco; 2 School of Medicine, University of California, Davis; 3 Storywalkers Consulting, Davis, California; 4 Division of Population Behavioral Health, UCLA Health, Los Angeles, California

## Abstract

**Importance:**

The murder of George Floyd in 2020 spurred an outpouring of calls for racial justice in the United States, including within academic medicine. In response, academic health centers announced new antiracism initiatives and expanded their administrative positions related to diversity, equity, and/or inclusion (DEI).

**Objective:**

To understand the experiences of DEI leaders at US allopathic medical schools and academic health centers, ie, the structure of their role, official and unofficial responsibilities, access to resources, institutional support, and challenges.

**Design, Setting, and Participants:**

This qualitative study used key informant interviews with participants who held formal DEI positions in their school of medicine, health system, or department. Interviews were conducted from December 2020 to September 2021. Transcripts were coded using a phenomenographic approach, with iterative concurrent analysis to identify thematic categories across participants. Data were analyzed from January to December 2021.

**Exposure:**

Formal DEI role.

**Main Outcomes and Measures:**

Questions elicited reflection on the responsibilities of the role and the strengths and challenges of the unit or office.

**Results:**

A total of 32 participants (18 of 30 [56%] cisgender women; 16 [50%] Black or African American, 6 [19%] Latinx or Hispanic, and 8 [25%] White) from 27 institutions with a mean (range) of 14 (3-43) years of experience in medical education were interviewed. More than half held a dean position (17 [53%]), and multiple participants held 2 or more titled DEI roles (4 [13%]). Two-thirds self-identified as underrepresented in medicine (20 [63%]) and one-third as first generation to attend college (11 [34%]). Key themes reflected ongoing challenges for DEI leaders, including (1) variability in roles, responsibilities, and access to resources, both across participants and institutions as well as within the same position over time; (2) mismatch between institutional investments and directives, including insufficient authority, support staff, and/or funding, and reduced efficacy due to lack of integration with other units within the school or health system; (3) lack of evidence-based practices, theories of change, or standards to guide their work; and (4) work experiences that drive and exhaust leaders. Multiple participants described burnout due to increasing demands that are not met with equivalent increase in institutional support.

**Conclusions and Relevance:**

In this qualitative study, DEI leaders described multiple institutional challenges to their work. To effectively address stated goals of DEI, medical schools and academic centers need to provide leaders with concomitant resources and authority that facilitate change. Institutions need to acknowledge and implement strategies that integrate across units, beyond one leader and office. Policymakers, including professional organizations and accrediting bodies, should provide guidance, accountability mechanisms, and support for research to identify and disseminate evidence for best practices. Creating statements and positions, without mechanisms for change, perpetuates stagnation and injustice.

## Key Points

**Question:**

What are the experiences of leaders in diversity, equity, and inclusion (DEI) at US academic health centers?

**Findings:**

In this qualitative study, 32 leaders described a considerable range of expected responsibilities. Institutional resources rarely matched stated goals, with limited use of evidence or standards, and participants from marginalized backgrounds expressed strong motivation coupled with exhaustion from the mismatch between demands and support.

**Meaning:**

The findings of this study suggest that leaders charged with promoting diverse, inclusive, and equitable environments in academic health centers would benefit from direct investment in their efforts, guidance from policymakers and organizations, and assessment and dissemination of best practices.

## Teaser

This qualitative study reports on how diversity, equity, and inclusion (DEI) leaders at US medical schools and academic health centers perceive their roles and responsibilities, access to resources, and institutional challenges.

## Introduction

Following the murder of George Floyd, leaders in academic medicine announced new initiatives related to advancing diversity, equity, and inclusion (DEI). The Association of American Medical Colleges (AAMC) found that the percentage of institutions with dedicated DEI staff and offices rose from 75% in 2018 to 97% in 2021.^1^ Nearly all (96%) reported having a senior-level DEI administrator (eg, assistant dean, chief diversity officer).^1^

Despite the growth in DEI efforts, evidence on the expectations of, support for, or standards for DEI in academic medicine remains limited. A 2015 AAMC survey of DEI leaders found that more than half held a dean title, although their positions varied across units, from student and faculty affairs to community engagement and outreach.^1^ Most reported they had less than 50% of their time allocated to DEI work, with budgetary support ranging from $0 to $1 million. Their main priorities included student diversity, meeting Liaison Committee on Medical Education (LCME) diversity standards, institutional climate, and culturally competent care.^2^ In a smaller 2018 survey, most focused on student recruitment and retention.^3^ The LCME and the Accreditation Council on Graduate Medical Education (ACGME) have instituted diversity-related accreditation standards but not specifically for DEI administrators. The AAMC Group on Diversity and Inclusion published a toolkit to support new DEI leaders, and the National Association of Diversity Officers in Higher Education developed professional standards, but it is unclear whether academic medicine institutions incorporate these guidelines for diversity leaders.^4,5,6^

Researchers in higher education have found that diversity initiatives are often broad in scope, unclear in meaning, and limited in their effectiveness. Following legal and political constraints on affirmative action policies, university leaders embraced the cause of diversity rather than racial justice.^1^ Subsequently, universities more often tasked diversity administrators with the discussion of diversity, including publishing statements and missions, rather than pursuing meaningful systems change.^5^ Furthermore, repeatedly launching new initiatives can displace substantive reforms by rearranging priorities and failing to invest in existing work.^6^ DEI leaders are often scattered in silos that preclude collaboration and communication. To work effectively, DEI leaders require equity-focused directives, sustained investment, organizational authority, coordination across units, and a commitment to reforming existing institutional structures.^7,8,9^

Our objective for this study was to describe the experiences of leaders in US academic medicine who have a formal DEI (or similar) position, particularly in the context of the renewed calls for attention to racial justice in medicine. We conducted key informant interviews to explore participants’ motivations, responsibilities, and their experiences in conducting DEI work. In the absence of a clear understanding of what DEI leaders can or should do, academic medicine cannot evaluate the impact of these initiatives. Consequently, schools and health systems run the risk of expending opportunities without advancing structural change.^10^ By exploring perspectives from DEI leaders, we can offer insights on strategies to support their success.

## Methods

We used the Standards for Reporting Qualitative Research (SRQR) reporting guideline to prepare this manuscript. The protocol was approved by the institutional review board of the University of California, Davis. We provided participants with a letter of information upon scheduling the interview, reviewed the document with participants, and obtained verbal consent.

### Researcher Characteristics and Reflexivity

The lead author (C.J.E.) identifies as a Chicane nonbinary medical student at the time of the study, who has created and participated in institutional DEI and racial equity initiatives. The primary interviewer (M.S.) identifies as a White cisgender man and professional facilitator with expertise in diversity in higher education. Coinvestigator (E.B.) identifies as a Black cisgender woman physician researcher with expertise in racism in medical education and currently serves as a senior DEI leader for an academic medical center. The senior investigator (M.K.) identifies as an Asian American cisgender woman researcher who has served in multiple DEI positions. Coinvestigator (M.R.L.) identifies as a White cisgender woman medical student. The collective team background and experience fostered planning, analysis, and discussion from multiple perspectives. Our status as a multiracial, multi-ethnic team enabled inquiry and analyses that included racial and ethnic positionality-specific examination.

### Approach

We used a phenomenographic approach to construct a representation of the variation in nature, positionality, and experience of those with DEI roles. We analyzed our topic within the multilayered context of academic medical institutions.^11^

### Recruitment and Sampling

We recruited participants from US medical schools and academic medical centers who held, or recently held, formal leadership roles in an office of Diversity, Equity, and/or Inclusion, which referred to all administrative units whose primary intent is to foster 1 or more of these goals at their respective program or institution. Although we used the term offices, we noted potential participants held titled roles over many types of structures, eg, committee, division, or center.

At the time of the study, there was no national directory of DEI leaders, and as noted previously, this population was rapidly changing. We conducted initial recruitment via email through professional networks, the UC Davis Center for a Diverse Healthcare Workforce, and contacts from prior studies conducted by the team. We supplemented with snowball sampling and purposive sampling to ensure we obtained perspectives from different geographic regions (West, Midwest, South, and Northeast) and types of institutions (public and private; schools and health systems). Participants were assigned an alphanumeric study identifier (ID) at recruitment.

### Data Collection

We conducted video interviews from December 2020 to September 2021. Interviews lasted approximately 1 hour and were digitally recorded and transcribed using Zoom services. We labeled speakers with participant study ID prior to recording and labeled files by study ID. Two authors (M.R.L. and C.J.E.) then reviewed transcripts to correct errors and remove identifying information, including regional (eg, city, county, and state), institutional, and programmatic details.

No interviews were discarded. We were unable to collect data on nonparticipants, other than name and institution, so we were unable to discern patterns of nonparticipants related to individual characteristics.

Critically, we conducted interviews early in the COVID-19 pandemic, shortly following the murder of George Floyd in the summer of 2020. Therefore, our data and analyses reflected the co-occurrence with these events. Discussions explored DEI structures (eg, roles, placement within institutions, formal resources, and level of influence) as well as participants’ personal experiences (eg, motivations, challenges, and emotional hurdles). The full interview guide is provided as the eAppendix in Supplement 1.

### Data Analysis

We used a phenomenographic approach with concurrent analysis to identify thematic categories across participants, rather than a focused analysis of singular experiences. Two authors conducted preliminary independent review of 5 transcripts (C.J.E and M.K.) to identify initial categories. Following review and full team discussion, 1 author (C.J.E.) reviewed the remaining transcripts to further develop and revise codes. In instances of differences of opinion, the team discussed and developed an agreed-on code and/or set of codes for the lead author to review and apply. The full team reevaluated the subsequent coding structure an additional 2 times, and then 1 reviewer (C.J.E.) conducted the final analysis and coding to produce final themes. We used Dedoose version 9.0.107 for initial code development, followed by hand-coding of transcripts to produce final themes. To enhance trustworthiness, we created an audit trail of detailed memos and used investigator triangulation of experiences in DEI roles for verification and context.

## Results

Our final sample consisted of 32 participants (18 of 30 [56%] cisgender women; 16 [50%] Black or African American, 6 [19%] Latinx or Hispanic, and 8 [25%] White) from 27 institutions (Table 1). More than half held a dean position (17 [53%]), and several held 2 or more DEI roles (4 [13%]). Two-thirds identified as underrepresented in medicine (20 [63%]) and one-third as first generation to attend college (11 [34%]). Regional distribution somewhat favored the eastern United States, with more than one-third of participants in the South.

**Table 1.** Participant and Institutional Characteristics

Characteristic	No. (%)
**Participants (n = 32)**	
Gender^a^	
Cisgender woman or female	18 (56)
Cisgender man or male	12 (44)
Race and ethnicity^a^	
Asian or Asian American	3 (9)
Black or African American	16 (50)
Latinx or Hispanic	6 (19)
White	8 (25)
Self-descriptors^b^	
Underrepresented in medicine	20 (63)
First generation to attend college	11(34)
Socioeconomically disadvantaged background	8 (25)
Rural background	2 (6)
Experience, mean (range), y	
In current role	3 (0.1-10)
At institution	14 (1-39)
Of diversity work	15 (4-50)
In medical education	14 (3-43)
**Institutions (n = 27)**	
Type	
Public	19 (70)
Private	8 (30)
Region	
South	10 (37)
Northeast	6 (22)
Midwest	6 (22)
West	5 (19)

^a^ Self-identified by participants in open-ended response. Race and ethnicity have been aggregated into 4 categories to protect confidentiality. Two respondents chose not to answer regarding gender. No respondents identified as trans, nonbinary, gender nonconfirming, or other minoritized gender identity. All responses were counted for those with multiple racial and ethnic identities, resulting in a total number greater than the number of participants. No participants identified as American Indian, Alaska Native, Native Hawaiian, Pacific Islander, or as a member of other Indigenous groups.

^b^ Self-identified by participants. Responded yes to 1 or more of the following: “Please indicate any terms that describe you: socioeconomically disadvantaged, first generation to attend college, disability, rural, underrepresented in medicine (URiM).” No participants reported being disabled or having a disability.

### Theme 1: Scope, Expectations, and Resources

Participants described responsibilities spanning clinical to biomedical to public health areas (Table 2). All reported formal goals of increasing workforce diversity and improving institutional climate. Many shared overlapping objectives, such as increasing capacity, broadening reach, and building credibility.

**Table 2.** Major Themes of DEI Leaders’ Experiences

Theme and subtheme	Supporting quotes (participant)
**Theme 1: scope, expectations, and resources**	
Autonomy combined with insufficient expectations or guidance	“When I see that [the dean] has given me full rein, it’s great, but it’s also of course it’s tough, because -when do I start? How do I start? Wait, what do you want me to do?… Give me some kind of guideline.” (ID22)
	“It can be a tad overwhelming and kind of scary—what if I don’t do this right? I’m drinking out of a fire hose… [there’s no one who gives] clear cut goals or objectives. Leadership would theoretically know those… but they are not giving out instructions.” (ID03)
Rising informal, on-demand expectations	“I feel like a lot of the energy that I would have been placing toward the students and the residents has gone toward the faculty and really putting out fires.” (ID62)
	“There really has been an explosion of work… with the events of last year. It’s a very open-ended responsibility and we are the go-to people. I’m not paid and my co-chair’s not getting paid, so it’s a bit challenging.” (ID18)
Expectations expanded due to complex reporting structures	“One situation required me to talk to the dean, the chair of the department, the faculty, and it will probably create at least five new meetings for me, that could be time I could be spending in other ways.” (ID06)
	“I exist in several parts of our organizational chart. In some cases, it looks like I’m reporting to myself.” (ID94)
Volatility in leadership commitment over time	“Honestly, it was very frustrating, because I’ve been screaming about this for 15 years, and last year happened and people were going ‘Oh, my god.’… I’m terrified it’s going to go away and that people are going to lose focus.” (ID228)
Variability in expectations increases vulnerability for underrepresented leaders	“Usually these positions are held by underrepresented minorities, but then when the work doesn’t get done then you can blame them.… We put so many expectations, and if there is a problem, it’s the person of color who didn’t do it, or did it wrong. It can be a setup and I think that people need to be careful about that.” (ID227)
**Theme 2: institutional expectations and investment**	
Inadequate tangible resources	
Staff	“So, the dean’s office... come back next year say oh… how come you didn’t do it? Because we have nobody dedicated to make that happen.” (ID02)
Budget	“It would be good to have a defined budget.… We do a lot with very little, sometimes [my] credit cards, and the school reimburses really slowly.” (ID83)
Individual time allocation	“On paper I’m supposed to be 65% (DEI) role… so I’ve slowly ramped up from 30% to 150%.” (ID62)
	“On my paperwork, 20% for the Associate Dean…. In truth is my job is 50% teaching, 50% research, 50% administration, 50% service.” (ID94)
Inadequate intangible resources	
Authority	“The committee really doesn’t have… much authority [or] resources to make things happen. But the dean’s office is happy because as a committee they could say, ‘I’ve given it to the committee.’” (ID02)
	“It’s very much leadership by influence rather than by mandate, so… not as though they can really steer the directive.” (ID14)
Placement in organizational hierarchy	“I wish I worked within a larger department, with the division chiefs, because they’re the ones who are really calling the shots with the hires.… I can hold the chairs accountable by collecting their data, but the chairs really have to hold their division chiefs accountable.” (ID48)
	“It’s also extremely political. The community wants to see the words ‘white supremacy,’ ‘anti-racism,’ up front in bold and the institution wants to say, ‘Can we all just get along?’ There is the real rub. What the people above me want me to say and what the people below me really feel they need to hear.… I have yet to get the people above me to truly understand the magnitude of the problem.” (ID207)
Undermined trust in institution	
DEI position or office as substitute for institutional change	“Even this new elevation, if you don’t put a true commitment to that… I feel it is completely symbolic.” (ID224)
	“It’s almost as if they deferred all issues—okay, we got her so we’re no longer responsible for anything.” (ID11)
	“There’s this clamoring for activities, statements and webinars, which in the big picture may or may not address the real concerns- implicit bias, structural racism- which are embedded in our in our academic world. That’s going to take much longer. It’s not going to be an hour webinar and then all sudden okay we’re done.” (ID229)
Devaluation of DEI work relative to other areas	“There’s no team, there’s no operational budget… there was no thinking behind, how do we move forward.… If we were talking about [a comparable initiative in] research, or if we’re talking about clinical care—that wouldn’t happen.” (ID34)
	“A lot of times people come into [the DEI] space and they are viewed by many other faculty as individuals who quote unquote ‘couldn’t make it in clinical practice or academia….’” (ID02)
**Theme 3: evidence-based frameworks, theories of change, or standards of expertise**	
Reactive, rather than proactive, directives	“Now that we have this awakening from a horrific murder last year, no one wants to be on the wrong side of history. Are we putting a Band-Aid on something, and are we just being reactionary again versus being intentional and proactive in our efforts, that it is sustainable over time.” (ID34)
Need for frameworks and theory on institutional change	“Even if we just looked at best practices, that will be extremely valuable for other people. It would also be important for us going forward as a quality issue, what works, what doesn’t work, what’s worth our investment both time and financially going forward.” (ID03)
	“This work, it’s really hard and there are no experts. If we knew how to do this, it would have been solved and addressed already so we wouldn’t be having this conversation… I’ve been doing this work for 40 years. It’s challenging work. It’s challenging in the sense that that nobody knows how to do it.” (ID16)
Lack of consistent evidence-based measures for tracking and reporting DEI work	“We have the LCME accreditation process so that’s the yardstick that I will be measured by... the way I look at it that’s a pretty low barrier to reach.” (ID03)
	“That’s one of the mistakes that we made in this, in diversity… we believed in kumbaya moments, things that make people feel good but weren’t sustainable. Surprisingly as an academic medical center, we didn’t do this as a scholarly project.” (ID06)
	“If it comes on the chopping block and go to the powers that be, whether it be the state or campus… [we want to be able to] say ‘Listen, we have these great outcomes, we’re going to lose this,’ so that’s why it’s really important that we have metrics to capture this data.” (ID75)
Lack of training and standards in expertise in academic medicine	“Being an MD doesn’t mean you have any training on diversity issues, on critical race theory, critical gender theory, history of race in America.” (ID228)
	“Most of the faculty in medicine they’re not trained on this, they’re not prepared to do this... because seeing patients does not mean that you’re doing DEI work. You know, sometimes you’re contributing to the problem actually.” (ID236)
**Theme 4: personal impact of DEI work**	
Motivated by personal experiences of harm	“… they’re not terribly many people like myself in the halls that I walk down on a daily basis. But I understand the importance of my own presence in the territory that I inhabit, and try to share the values that I have developed over time with others, in a way that’s not combative but hopefully compelling.” (ID83)
	“[Students] may see me as this polished, put-together professional who might not have these issues, but yeah, it’s me when I get pulled over. I keep my hands, right here too.” (ID71)
Emotional consequences and burnout	
Isolation	“The obstacle continues to be, it was lonely then, and it is better now, but it’s still hard to be one of only.” (ID229)
Demands of supporting marginalized groups	“There’s a lot of academic trauma... students are... having to code switch and send their representative to school and work every single day.” (ID62)
Lack of opportunity or recognition of personal impacts	“Exhausting. Absolutely exhausting for multiple reasons. My job is to have hope, my job is to be there to absorb for everybody else, my job is to figure out what I can do without after every kaboom to help the community heal... But [I] don’t have time to heal or time to process.” (ID207)

Abbreviations: DEI, diversity, equity, and inclusion; ID, identifier; LCME, Liaison Committee on Medical Education; MD, medical doctor.

Responses indicated a broad range in authority, leadership endorsement, and resources, even when participants held seemingly similar roles. For example, among 3 participants with associate dean titles, ID62 reported 20% full-time equivalent (FTE) allocation to chair the antiracism task force and conduct oversight of medical school metrics, training, and policies, with 4 staff and a $1 million budget. ID11 had 30% FTE to oversee undergraduate medical school curriculum as well as recruitment and retention of trainees, faculty, and department chairs, with no staff or budget. Meanwhile, ID94 described their time as 20% or 50%, depending on their leadership’s perspective, and supervised undergraduate pathway programs, curriculum, LCME and ACGME accreditation, and faculty diversity, with the assistance of 1 staff coordinator and federal grants. Those working at the department level had no staff and minimal compensation and funding.

Many reported a high level of autonomy, but nearly all preferred clearer, more structured expectations from leadership (Table 2). Participants regularly juggled informal on-demand requests, such as providing emotional support following incidents of discrimination and crafting rapid institutional response statements. They described how complex reporting structures (eg, reporting simultaneously to school, health system, and university leadership) required them to expend time and energy developing and maintaining a broad network of connections.

Participants with longer tenure described institutional volatility in commitment and resources. Many reported that expectations increased following the renewed racial justice movement but questioned whether these changes merely reflected a temporary institutional response vs sustained effort. Furthermore, institutions hired underrepresented individuals for DEI positions; this offered needed perspective but placed these individuals at heightened vulnerability: DEI failures could also be blamed on leaders from marginalized racial, ethnic, income, or gender groups.

Two participants (ID48 and ID62) expressed optimism because they had received new directives with clear expectations, appropriate staffing and budget, and institutional accountability. Their institutions required department chairs and division chiefs to prepare detailed faculty DEI plans and accountability incorporated into performance reviews, signaling DEI as an institutional priority. The DEI office provided data and programming support for these leaders, positioning the DEI administrator as a collaborative, rather than adversarial, partner.

### Theme 2: Institutional Directives and Investments

Participants consistently described insufficient investment relative to institutional expectations (Table 2). Investment consisted of both tangible resources, eg, budget, staff, time, and compensation, as well as intangible resources, such as authority and leadership endorsement. Most felt that senior leadership did not understand the level of support needed to pursue DEI goals effectively. Participant ID224 summarized, “They have high expectations, and the reality is that I am only one person.… Diversity is seen as compliance accreditation. [If] you’re really interested in changing the landscape… then you have to put money where your mouth is, to move the needle.”

Furthermore, participants had no consistent positioning within their organizations and often occupied a place in parallel to (rather than integrated in) main organizational units (eg, medical education, faculty development). Without the ability to directly guide operations, participants struggled to meet expectations. One participant (ID207) explained that they “do not have true power” but rather “power… by proxy.” Many characterized their institutional leadership as verbally supportive without concomitant sponsorship, thus hampering their abilities to advance organizational accountability (Table 2).

The mismatch between institutional expectations and resources, particularly relative to investments in research and clinical activities, undermined participants’ trust in their institution’s commitment to DEI. They questioned whether they played superficial, rather than substantive, roles. One (ID11) worried that the creation of the DEI office allowed their institution to absolve other units of accountability. Institutional devaluation further amplified racial prejudice from peers and staff, who perceived DEI leaders as less capable.

### Theme 3: Evidence-Based Frameworks, Theories of Change, and Standards of Expertise

Participants felt that the absence of structured institutional expectations increased the difficulty of their work (Table 2). They perceived that leadership frequently operated by reactionary response to an event, such as LCME accreditation or a major news crisis, that resulted in “clamoring for activities, statements, and webinars” rather than addressing “real concerns [such as] structural racism” (ID239). Participants noted the lack of root-cause analysis also contributed to the perception of institutional DEI as performative rather than substantive. As ID236 explained, DEI work required new expertise—otherwise, there would be no need for change. Several endorsed “that nobody knows how to do it” and wished for more scholarship on DEI practice in academic medicine.

The limited evidence base contributed to inconsistency in measurement and accountability. Participants expressed uncertainty around how to demonstrate success, which undermined their confidence and ability to advocate for DEI to institutional leadership. Some perceived LCME and ACGME accreditation as diversity compliance, but others felt the threat of losing accreditation at least motivated small steps toward reform.

Without a strong base of theory and scholarship, participants described a vacuum in expertise to assess qualifications for DEI positions. Participants explained that given that the majority of academic medicine faculty are physicians, most lack training on organizational development theory, implementation science, and historical and current systems of oppression in medicine. Instead, ID236 reflected, the lack of physicians with appropriate training contributes to the problems that DEI work is supposed to fix.

### Theme 4: Personal Impact of DEI Work

Participants referenced personal sources of motivation, often arising from their own lived experiences with tokenism, discrimination, and mistreatment in academic medicine (Table 2). Thus, despite the challenges described in the preceding themes, they held a strong commitment to support trainees and faculty from marginalized groups. As ID83 noted, “I understand the importance of my own presence in the territory, and try to share the values that I have developed over time with others, in a way that’s not combative but hopefully compelling.” Some reported leveraging their own marginalized identity to educate peers and leadership, putting a “personal spin on it” for people “to see the humanness.”

Many reported that the combination of personal and institutional marginalization contributed to deep professional isolation. Their DEI roles required them to serve as the face of their institutions in managing internal incidents of interpersonal discrimination and abuse yet also supporting trainees and peers. They also described the toll of leading institutional responses following highly publicized cases of racial and gender-based violence, while being chronically undersupported and unrecognized for their emotional labor. Participants reported growing burnout from the pressure of navigating the narrow space between institutional and community demands. Participant ID207 shared, “My job is to have hope, to absorb for everybody else, figure out what I can do after every kaboom to help the community heal…. But [I] don’t have time to heal or process [myself].”

## Discussion

Our findings offer insights into the experiences of DEI leaders in academic medicine during a period of heightened attention to racial injustices. The variability in roles and institutional investment reveal an overall lack of clarity on the aims and implementation of DEI initiatives. Furthermore, DEI work can occur at high personal cost to the individuals tasked with carrying it through.

Our findings are consistent with earlier studies that have found high variability in titles, scope, role, authority, and resources.^3,12^ The ambiguity of DEI roles and responsibilities, and the mismatch between expectations and investment, are emblematic of *decentralization,* described by sociologist James Thomas as: “1) lack of/slow coordination; 2) absence of regulations and/or enforcement; 3) unresponsiveness; 4) poor observational capabilities; 5) shared belief that no matter what organizational actors do, the same outcome persists.”^6^ Our participants’ guarded perceptions of new antiracism initiatives reflect the concern that academic medicine will remain unchanged. Recognizing this pattern is crucial, because some may conclude that the lack of progress suggests that DEI work cannot, or should not, be done.

Participants believed their challenges arose partly due to the absence of theories of change within academic medicine. Institutional leaders reference business and management fields to pursue clinical and educational reforms, but not for DEI work.^12^ For example, the field of implementation science applies organizational behavior knowledge, yet no participants reported using implementation science in DEI. This may reflect institutional oversight, but diversity scholars argue that the overall lack of strategy is a feature, rather than a bug, in the system.^7,8^ Under these conditions, DEI leaders run the risk of being perceived as ineffectual, and their own work as performative.^5^

Our participants expressed exhaustion and burnout from compensating for DEI decentralization, which was further amplified participants from minoritized racial and ethnic groups by staging difference: “the constant push for new programs, managed by men and women of color,” to signal a “new” effort by the university.^6^ Feminist scholar Sara Ahmed^5^ raised the cautionary warning that racially and ethnically minoritized DEI professionals then embody DEI for the institution, at a steep cost to their personal health. Black DEI professionals are particularly exploited by institutions to address systemic problems (without systemic resources), serve as a buffer between leadership and their constituents, and use their own identity as a stand-in for entire communities.^13,14^ Our participants detailed the fatigue that arises when they selectively express and repress aspects of their identities, without the structural or material support to change the very institutions that continue to harm them. Thomas^6^ cautions that “rather than addressing structural inequalities, the performative culture of diversity reproduces and exaggerates them.” Staging difference reproduces DEI leadership churn, leading to failed initiatives and repeated calls for minoritized workers to lead the next new initiative.

Our study offers rich detail on how DEI leaders may be stymied in academic medicine; however, they also suggest opportunities for change. First, organizations such as the AAMC can support developing specific guidelines on DEI objectives and resources and invest in successful programs. Second, the LCME and the ACGME can institute detailed DEI standards, including requirements for clear expectations, measurement, and institutional investment in monitoring and evaluation.^15^ The current diversity and inclusion accreditation standards created a theoretical structure for assessment, but measurement and evaluation remain vague and limited in fostering accountability.^16,17^ Third, institutions can establish professional expectations for DEI leaders, such as training and experience in organizational behavior, power assessment, critical race theory, and historical understanding of structural inequities. Fourth, DEI leaders must be granted resources—including staff, budgets, and authority—concomitant with their objectives and scope.

These recommendations may be particularly challenging to enact as policymakers in multiple states have terminated DEI funding, programming, and positions. The current climate further demonstrates the need for greater specificity and understanding of DEI objectives in academic medicine. DEI work that is valued only for its appearance, and not the design and impact, cannot produce the changes necessary to create diverse, equitable, or inclusive systems of care for our communities.

### Limitations

The limitations of this study include the timing, in a period when medical institutions and organizations increased DEI resources.^18^ However, as higher education and health care institutions experience growing staff shortages and public attention to racial justice wanes, institutional investment may be declining. Second, our recruitment process and stated aims may have selected for participants who were motivated to participate due to their personal challenges at work. As a qualitative study, we aimed for range and depth of experiences, rather than generalizability to the entire academic medicine population. We could not recruit from a national directory of DEI offices, leaders, or administrators in academic medicine. In 2018, Chen et al^3^ identified 112 offices in 148 allopathic schools, but since then, institutions have created multiple offices and positions within schools and across departments. Third, we focused on those with formal titles, but effective DEI initiatives rely on the broader campus community, so our findings do not capture the experiences of other important actors. We interviewed only 7 participants with departmental-level roles, and thus may not sufficiently capture how these positions have expanded recently; systematic documentation is needed.

## Conclusions

In this qualitative study, DEI leaders described multiple institution-level challenges to their work, including limited resources, unclear expectations, and a lack of evidence-based practices. While the transformation of academic medicine is long overdue, dismantling systems requires large-scale, sustained investment, grounded in theories of change, supported by evidence, and constantly interrogated for purpose, operationalization, and impact. Relegating the work to a handful of siloed individuals can set DEI leaders up for burnout and perceived failures. Recent events—from litigation against affirmative action to states’ efforts to eliminate DEI offices and related work on university campuses—reflect societal pushback against the (small) gains of the racial justice movements of 2020.^19,20,21^ Sustained commitment to health equity, including the training and membership of the medical profession, is more important than ever.

## Notes

Supplement 1. **eAppendix.** Interview Guide

Supplement 2.
Data Sharing Statement
