# Supplementary material for: Experiences of Leaders in Diversity, Equity, and Inclusion in US Academic Health Centers
Source: JAMA Netw Open. 2024 Jun 13;7(6):e2415401. doi: 10.1001/jamanetworkopen.2024.15401 (PMC11177162; doi:10.1001/jamanetworkopen.2024.15401)
Supplement: Supplement 1. — eAppendix. Interview Guide [file jamanetwopen-e2415401-s001.pdf]

Supplemental Online Content

Esparza CJ, Simon M, London MR, Bath E, Ko M. Experiences of leaders in diversity, equity, and inclusion in US academic health centers. *JAMA Netw Open.* 2024;7(6):e2415401. doi:10.1001/jamanetworkopen.2024.15401

eAppendix. Interview Guide

This supplemental material has been provided by the authors to give readers additional information about their work.

© 2024 Esparza CJ et al. *JAMA Network Open.*

eAppendix. Interview Guide

Discussion Guide- Leadership:

Thank you again for taking the time to participate in our study. As we discussed, our aims are to describe the Offices of Diversity, Equity and Inclusion in U.S. medical schools and develop understanding of – the experiences of those who work in these spaces. In this interview, we are asking for your personal reflections, perceptions and opinions – even if it differs from the perspective or presentation of your institution.

Please note that this interview will be recorded and transcribed. Your participation is voluntary: you can choose not to answer a question, or you can withdraw at any time. We take the confidentiality of your responses very seriously. You are not expected to state any names, including your own and those of colleagues, your institution, and/or location. We will delete identifying information from the transcript before your data are analyzed and coded.

1. **First, can you tell us about your role related to diversity, equity and inclusion at your institution?**
    - *What is your position or title?*
    - *How long have you been in that position?*
    - *Can you describe how you came to work in this position?*
    - *How long have you been at your current institution?*
    - *Do you receive a stipend or compensation for your role in DEI?*

2. **Now, we are going to ask you a little about your office (or department, division, unit). Can you please describe what is the mission of your office?**
    - *When did the office/department/division/unit come into being?*
    - *What events or decisions led to the creation of the office/department/division/unit?*
    - *How does your institution define diversity, equity and inclusion? (Interviewer: modify according to title, e.g. "diversity," or "diversity and inclusion," and so on)*
    - *Can you explain who defined this mission? To your knowledge, has it changed over time, and if so, why?*
    - *Do you feel you have a clear understanding of the mission of your office?*
    - *What role, if any, does LCME or ACGME accreditation play in the operationalization of your school's DEI goals/mission?*

3. **Can you describe where your office 'sits' in relation to other units in the school?**
    - *Who holds your office accountable for DEI outcomes?*
    - *What other units in the school have you, or are you, currently working with?*

4. **What are the main activities of your office?**
    - *Can you describe what types of services/programming you offer or oversee?*
    - *How many people (or positions) are working in your office?*
    - *Can you describe who/how the services to be offered are selected and supported?*
    - *Do you feel you have a clear understanding of what services/programming is expected of your office?*
    - *What role, if any, does LCME or ACGME accreditation play in your school's DEI services and programming?*
    - *To your knowledge, what, if anything, has changed in your office/department/division/unit over the past 10 years?*
        - *For former leadership: What has changed since you left the office/ department/division/unit?*
    - *Has the office/department/division/unit changed in recent years?*
        - *Priorities/direction?*
        - *Trainings*
        - *Initiatives/New Projects*
        - *Staffing/resources?*
        - *Level of authority and visibility?*

5. **Now we would like ask about your experiences in conducting DEI work at your school. First, can you please describe what your responsibilities are, with respect to the office?**
    - *Describe how much is expected of you to conduct this work – is it full-time, part-time, voluntary?*
    - *Are you compensated or otherwise granted salary or time support in this position?*
    - *To whom/what leadership do you report in your school?*
    - *If participant responded part-time: Is this work recognized and rewarded on par with your other activities?*

6. **Second, what do you believe your unit/office does well in relation to DEI?**
    - *How do you define success for your unit and your diversity work?*
        - *Do the successes of your unit/office and the institution match?*
    - *What do you think has been most helpful to your success in DEI?*
    - *What types of support, whether financial, staffing, physical space, peer mentorship, and so on- have been provided by your school?*
    - *Does your unit collaborate with other DEI units (whether within your institution, in your region or nationally)?*
    - *How do you understand that medical schools nationally are in alignment related to DEI activities? What are the common forces shaping units like yours?*

7. **Finally, what types of challenges have you or your predecessors/successors encountered in advancing DEI at your school?**
    - *Can you describe a specific example or situation?*
    - *What do you think are the main drivers or sources of those challenges? Who are the facilitators of those barriers?*
    - *Do you feel that you/your office has the administrative authority to enact change?*
    - *Do you feel that you have the adequate resources- financial, staffing, physical space and materials- to conduct DEI work?*
    - *What would be most helpful to you, at this moment, to advance your school's DEI efforts?*

Thank you again for your time and participation. Do you have any other information or thoughts you would like to share? Additionally, please let us know if you have any colleagues at other institutions who may be interested in participating in this study.

© 2024 Esparza CJ et al. *JAMA Network Open.*
