# Supplementary material for: Experiences of Leaders in Diversity, Equity, and Inclusion in US Academic Health Centers
Source: JAMA Netw Open. 2024 Jun 13;7(6):e2415401. doi: 10.1001/jamanetworkopen.2024.15401 (PMC11177162; doi:10.1001/jamanetworkopen.2024.15401)
Supplement: Supplement 2. — Data Sharing Statement [file jamanetwopen-e2415401-s002.pdf]

Data Sharing Statement

Esparza. Experiences of Leaders in Diversity, Equity, and Inclusion in US Academic Health Centers. *JAMA Netw Open*. Published June 13, 2024.
doi:10.1001/jamanetworkopen.2024.15401

Data
Data available: No

Additional Information
Explanation for why data not available: Data collected for this study include information that pose personal and professional risks to participants if confidentiality is breached, and participants were assured that any potentially identifying information would remain available only to the research team.
